# Identification and validation of hub genes and potential drugs involved in osteoarthritis through bioinformatics analysis

**DOI:** 10.3389/fgene.2023.1117713

**Published:** 2023-02-09

**Authors:** Wenbo Xu, Xuyao Wang, Donghui Liu, Xin Lin, Bo Wang, Chunyang Xi, Pengyu Kong, Jinglong Yan

**Affiliations:** ^1^ The Second Affiliated Hospital of Harbin Medical University, Harbin, China; ^2^ Department of Pharmacy, Harbin Second Hospital, Harbin, China; ^3^ Department of Oncology, Heilongjiang Provincial Hospital, Harbin, China

**Keywords:** osteoarthritis, hub genes, bioinformatics analysis, GEO, ferroptosis, pyroptosis

## Abstract

**Purpose:** Osteoarthritis (OA) is a common degenerative disease, which still lacks specific therapeutic drugs. Synovitis is one of the most important pathological process in OA. Therefore, we aim to identify and analyze the hub genes and their related networks of OA synovium with bioinformatics tools to provide theoretical basis for potential drugs.

**Materials and methods:** Two datasets were obtained from GEO. DEGs and hub genes of OA synovial tissue were screened through Gene Ontology (GO) annotation, Kyoto Encyclopedia of Genes and Genomes (KEGG) pathway enrichment as well as protein—protein interaction (PPI) network analysis. Subsequently, the correlation between expression of hub genes and ferroptosis or pyroptosis was analyzed. CeRNA regulatory network was constructed after predicting the upstream miRNAs and lncRNAs. The validation of hub genes was undertook through RT-qPCR and ELISA. Finally, potential drugs targeting pathways and hub genes were identified, followed by the validation of the effect of two potential drugs on OA.

**Results:** A total of 161 commom DEGs were obtained, of which 8 genes were finally identified as hub genes through GO and KEGG enrichment analysis as well as PPI network analysis. Eight genes related to ferroptosis and pyroptosis respectively were significantly correlated to the expression of hub genes. 24 miRNAs and 69 lncRNAs were identified to construct the ceRNA regulatory network. The validation of EGR1, JUN, MYC, FOSL1, and FOSL2 met the trend of bioinformatics analysis. Etanercept and Iguratimod reduced the secretion of MMP-13 and ADAMTS5 of fibroblast-like synoviocyte.

**Conclusion:** EGR1, JUN, MYC, FOSL1, and FOSL2 were identified as hub genes in the development of OA after series of bioinformatics analysis and validation. Etanercept and Iguratimod seemed to have opportunities to be novel drugs for OA.

## 1 Background

Osteoarthritis (OA) is a common degenerative disease in middle-aged and elderly people all over the world, of which the prevalence has gradually increased due to the aging population and the trend of the overweight (Loeser et al., 2016; Roemer et al., 2022). According to the data from World Health Organization (WHO) in 2019, about 250 million people worldwide suffered from OA (Hunter and Bierma-Zeinstra, 2019). Synovitis is one of the most important pathological manifestations in the occurrence and development of OA, running through the whole process (Sharma, 2021). Studies have shown that synovial lesions usually occur earlier than cartilage, which can be detected by MRI in several patients with small joint injury on X-ray. In addition, low-grade synovitis contributes to radiographic and pain progression (Sanchez-Lopez et al., 2022). The pathological changes of synovitis are complex and diverse, leading to the lack of specific treatment for OA. Therefore, it is necessary to explore the pathogenesis and diagnostic markers of OA from the perspective of synovium to find the therapeutic targets of OA, alleviate symptoms and promote the prognosis.

Ferroptosis is a programmed cell death mode caused by abnormal oxidation, which is regulated by glutathione peroxidase 4 (GPX4), with characteristics of iron-dependent accumulation of lipid peroxide. Kennish et al. (2014) showed that the iron level in serum of OA patients was positively correlated with the severity of OA, suggesting the existence of abnormal iron homeostasis, but its effect in synovium has not been reported. Pyroptosis is another mode of programmed cell death, which occurs when pattern recognition receptors (PRRS) induce the activation of cystine aspartic protein 1 (caspase-1) or caspase-11, which is characterized by the destruction of cell membrane and the release of cytokines. Pyroptosis has been shown to be involved in synovits, that is, Interleukin-1β (IL-1β) in the inflammatory environment derives from synovium rather than cartilage (Borgonio et al., 2014).

Bioinformatics tools have been widely used to process microarray data to determine differentially expressed genes (DEGs) and conduct various analysis. In this study, combining with bioinformatics analysis and verification, we explored and screened the hub genes of OA synovial tissue, and discussed the correlation between them and ferroptosis or pyroptosisy, then constructed the upstream ceRNA regulatory network to evaluate the reliability of these genes as the prediction or treatment targets of OA. Finally, we identified and validated potential drugs targeting pathways and hub genes through Comparative Toxicogenomics Database (CTD), Drugbank and Drugs-Genes interaction (DGI) database.

## 2 Materials and methods

### 2.1 Data collection

Gene Expression Omnibus (GEO) (https://www.ncbi.nlm.nih.gov/geo/) was used as our data source (Barrett et al., 2013), where “Osteoarthritis” was entered as the keyword in the search box for detection. The selected data set included the gene expression array of osteoarthritis and normal synovial tissue of human samples. Finally, GSE55235 (GPL96 platform), GSE55457 (GPL96 platform) were determined as the data sets of this study.

### 2.2 Data preprocessing and identification of DEGs

The data of GSE55235, GSE55457 were downloaded with the format of MINiML. The mRNA expression data of OA and normal synovial tissue in each data set were analyzed by limma software package of R software (Yu et al., 2012). *P*-value was analyzed in GEO to correct false-positive results, which <0.05 and |log2 (fold change)| > 1 was defined as the threshold. DEGs were then obtained, and were visualized by volcano map and heat map. common DEGs were identified after the intersection of the two datasets.

### 2.3 Enrichment analysis of common DEGs and PPI network construction

Gene Ontology (GO) annotation of the common DEGs was performed by the Database for Annotation, Visualization and Integrated Discovery (DAVID, version 2021) (Sherman et al., 2022), including biological process (BP), cellular component (CC), and molecular function (MF). *p* < 0.05 was determined as a significant margin for all analysis. Column chart was plotted by “http://www.bioinformatics.com.cn,” a free online platform for data analysis and visualization. The Kyoto Encyclopedia of Genes and Genomes (KEGG) pathway enrichment analysis of the common DEGs was carried out by R package “clusterprofiler” (Version 4). Hypergeometric test was performed to evaluate the significance of pathway–pathway association: phyper (k-1, M, N-M, n, lower. tail = F). Metascape (http://metascape.org) was used for protein - protein interaction (PPI) network analysis of common DEGs (Zhou et al., 2019). PPI analysis was performed using the following databases: STRING, BioGrid (Kondo et al., 2019), OmniPath and InWeb_IM (Li et al., 2017). Physical score >0.132 was selected to be the standard of gene screening. In addition, molecular complexity detection (MCODE) was used to identify densely connected network components and obtain the hub genes of OA (Bader and Hogue, 2003). The network was visualized using Cytoscape.

### 2.4 Correlation between OA hub genes and ferroptosis and pyroptosis related genes

19 genes related to ferroptosis and 21 genes related to pyroptosis contained in microarray data were selected for correlation analysis with the hub genes (Liu et al., 2020a; Wu et al., 2021). Due to the small sample size of the three groups of data related to ferroptosis, the samples were combined and normalized (Zhang et al., 2019). The data was standardized using “normalize.quantiles” function in the preprocessCore package of R, and was evaluated through the box diagram. The batch effect of data was evaluated by comparing the visual PCA diagram before and after removal. The polygenic correlation map was displayed by pheatmap package of R. Spearman’s correlation analysis was used to describe the correlation between quantitative variables without normal distribution. *p* < 0.05 was considered statistically significant.

### 2.5 Analysis of ceRNA regulatory network of hub genes

ENCORI (Li et al., 2014) (https://starbase.sysu.edu.cn) and TargetScan (McGeary et al., 2019) (https://www. targetscan. org/vert_80/) were used to predict miRNAs which regulated hub genes. The first three reliable miRNAs were selected after the intersection of the prediction. LncBase database v3.0 (Paraskevopoulou et al., 2016) (https://diana.e-ce.uth.gr/lncbasev3/interactions) was used to predict lncRNAs which regulated miRNAs above. The first three reliable lncRNAs were selected for each miRNA. Finally, the ceRNA regulatory network of hub genes was constructed.

### 2.6 Extraction of human fibroblast-like synoviocyte (FLS)

The study was designed according to the Declaration of Helsinki, and was approved by Ethic Committee of the Second Affiliated Hospital of Harbin Medical University (KY 2021-256). Informed consent was obtained from each donor. Synovium of 3 OA patients (age 54–70 years, Kellgren-Lawrence grade 4) that underwent total joint arthroplasty (TKA) and 3 patients (age 56–68 years) that underwent meniscectomy without OA were obtained at the time after surgery. All patients were confirmed without Rheumatoid Arthritis, acute trauma, tumor or infection of knee joint. Briefly, synovium was cut into pieces at the final size about 0.5 mm*0.5 mm, and put into 0.1% type I collagenase (Biosharp, China, BS163). α-MEM medium (Cytiva, United States, SH30265.01) was added with 10% foetal bovine serum (ExCell Bio, China, FSD500) after 2 h. Primary cells could be seen climbing out after about 3–5 days. FLS of passage 6-8 (P6-8) were used in this study. 10 ng/ml of IL-1β (PEPROTECH, United States, 200-01B) were used to stimulate FLS of OA groups for 48 h in order to imitate the environment of OA, while complete medium was added into the FLS of control group. 10 μg/ml of Etanercept and Iguratimod (MedChem Express, China, HY-108847, HY-17009) were added along with IL-1β to FLS for the following test.

### 2.7 Screening of potential drugs for OA

CTD database (version 16766M) was used to search drugs targeting the KEGG pathways above. Drugs targeting hub genes were searched and compared using Drugbank (version 5.1.8) database and DGI database (version 4.2.0).

### 2.8 RT q-PCR

Trizol (Beyotime, China, R0016) was used to extract total RNA from FLS. After determining the concentration, 2 µg of total RNA was used to synthesize cDNA through cDNA synthesis kit. SYBR Green (ES Science, China, QP002) was used for qRT-PCR according to the instructions. The primer sequence of 8 hub genes and GAPDH were listed in Table 1. The mRNA level of a specific gene was calculated as 2-^ΔΔ^ Ct and normalized to GAPDH.

**TABLE 1:** Primers used for RT-qPCR amplification.

Gene	Primers	Sequence
ATF3	Forward	CCT​CTG​CGC​TGG​AAT​CAG​TC
	Reverse	TTC​TTT​CTC​GTC​GCC​TCT​TTT​T
EGR1	Forward	GGT​CAG​TGG​CCT​AGT​GAG​C
	Reverse	GTG​CCG​CTG​AGT​AAA​TGG​GA
FOSB	Forward	GCT​GCA​AGA​TCC​CCT​ACG​AAG
	Reverse	ACG​AAG​AAG​TGT​ACG​AAG​GGT​T
FOSL1	Forward	CAG​GCG​GAG​ACT​GAC​AAA​CTG
	Reverse	TCC​TTC​CGG​GAT​TTT​GCA​GAT
FOSL2	Forward	CAG​AAA​TTC​CGG​GTA​GAT​ATG​CC
	Reverse	GGT​ATG​GGT​TGG​ACA​TGG​AGG
JUN	Forward	TCC​AAG​TGC​CGA​AAA​AGG​AAG
	Reverse	CGA​GTT​CTG​AGC​TTT​CAA​GGT
JUNB	Forward	ACG​ACT​CAT​ACA​CAG​CTA​CGG
	Reverse	GCT​CGG​TTT​CAG​GAG​TTT​GTA​GT
MYC	Forward	GGC​TCC​TGG​CAA​AAG​GTC​A
	Reverse	CTG​CGT​AGT​TGT​GCT​GAT​GT
GAPDH	Forward	CAC​TCA​GAC​CCC​CAC​CAC​AC
	Reverse	GAT​ACA​TGA​CAA​GGT​GCG​GCT

### 2.9 Elisa analysis

The supernatant was collected and centrifuged at 1,000 × g at 4°C for 10 min, then was added to 96-well plates coverd by the antibody of each protein (Elabscience, United States). After incubated for 90 min at 37°C, Biotinylated Detection Ab was added to each well. After 60 min, the plates were washed for 3 times. HRP Conjugate was then added to each well and incubated for 30 min at 37°C in the dark. 90 μL of Substrate Reagent and 50 μL Stop Solution were then added. The optical density (OD value) of each well were determined at once by microplate reader at 450 nm.

### 2.10 Detection of intracellular ROS

FLSs of each group were seeded at a density about 2000 cells per well in 96-well plates. The medium was replaced by H2DCFDA (Biosharp, China, BL714A) working solution with the concentration of 10 μM. FLSs were then incubated at 37°C for 2 h in the dark and washed twice to fully remove the probes outside the cells. OD value of each well was determined at once by microplate reader at 525 nm.

### 2.11 Detection of lipid peroxidation

FLSs of each group were broken by ultrasonic cell crusher and centrifuged at 3,500 xg for 10 min at 4°C. MDA standard solution (Solarbio, China, BC0025) and the supernatant to be tested with chromogenic agent were boiled, centrifuged, and added to a 96-well plate respectively. The absorbance was measured at 450 nm, 520 nm, and 600 nm for detection by microplate reader. Then the concentration of MDA was calculated according to the instruction.

### 2.12 Cell viability assay

FLSs were seeded in 96-well plates at a density at about 2000 cells per well. After adding 10 ul of CCK-8 reagent (Beyotime, China, C0037) at 24 h or 48 h respectively, the plates were put into the incubator for 30 min away from light. The absorbance of each well was measured at 450 nm by microplate reader.

### 2.13 Statistical analysis

All the data of validation was presented as the means ± standard deviation (SD). Statistical analysis was performed using GraphPad Prism version 6.02. Differences in numerical data between two groups were determined by *t*-test, while four groups were determined by One-way ANOVA followed by a Bonferroni post-hoc test. *p* < 0.05 was defined as statistically significant.

## 3 Results

### 3.1 Data collection and identification of DEGs

**FIGURE 1:**
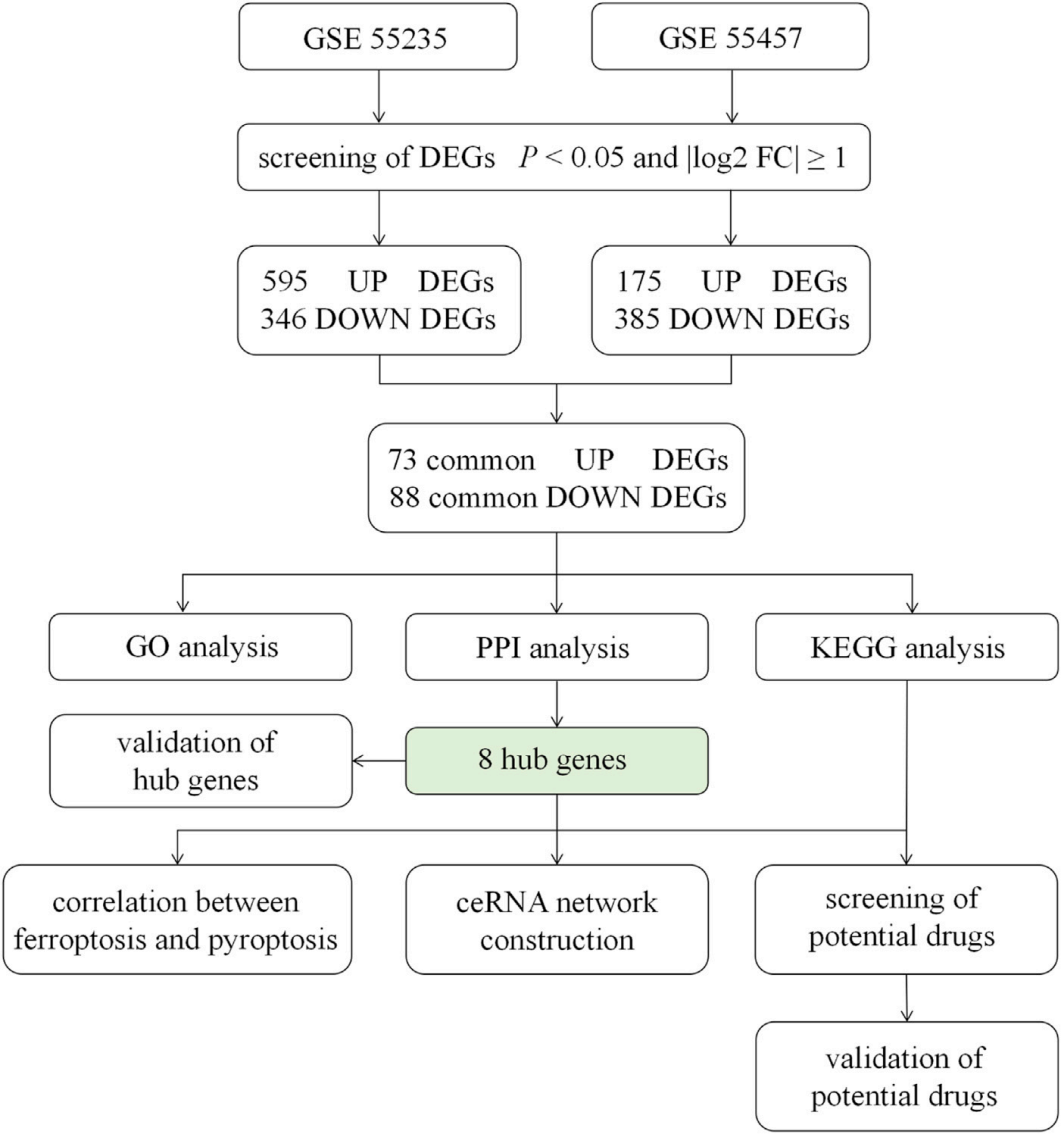
Flow chart of the study design. GSE55235, GSE55457 were determined as the data sets of this study, from where we screened DEGs by the standard of *p* < 0.05 and log2|FC| ≥ 1. After the intersection, 71 up-regulated DEGs and 88 down-regulated DEGs were obtained, followed by GO analysis, PPI network construction and KEGG pathway analysis. The correlation between hub genes and ferroptosis or pyroptosis and the construction of ceRNA were undertaken. Moreover, the expression of 8 hub genes was validated. Finally, potential drugs targeting pathways and hub genes were identified and validated. DEGs = differentially expressed genes; FC = fold change; GO = gene ontology; PPI = protein - protein interaction; KEGG = Kyoto Encyclopedia of Genes and Genomes; ceRNA = competing endogenous RNA.

**FIGURE 2:**
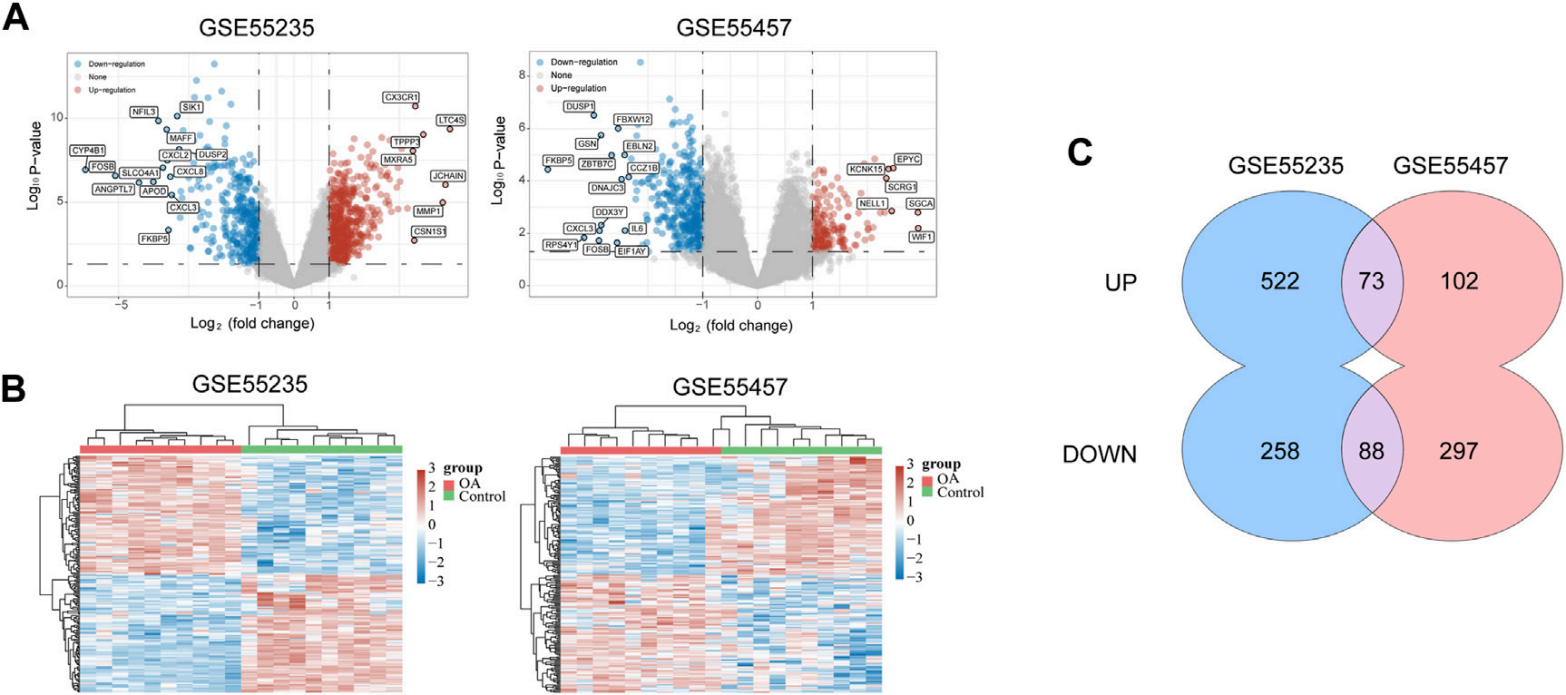
Data collection and identification of DEGs. **(A)** Volcano plot of DEGs in two datasets (*p* < 0.05 and log2|FC| ≥ 1). The red nodes represent up-regulated genes, and the blue nodes represent down-regulated genes. **(B)** Heat map of DEGs in two datasets, in which different colors represent different expression trend. Due to the large number of DEGs, the top 50 up-regulated and down-regulated genes with the largest FC were shown. **(C)** Venn diagram of two datasets which showed up-regulated and down-regulated genes respectively. DEGs = differentially expressed genes; FC = fold change.

The flow chart of the study design was showed in Figure 1. A total of 20 OA synovium samples and 20 normal synovium samples were obtained from two GEO database, including 10 OA samples and 10 normal samples from GSE55235 dataset and 10 OA samples and 10 normal samples from GSE55457 dataset. Relevant clinical data were not provided in the two datasets above. After screening the differential expression of mRNA between OA and normal synovium samples, 595 up-regulated DEGs and 346 down-regulated DEGs from GSE55235 as well as 175 up-regulated DEGs and 385 down-regulated DEGs from GSE55457 were obtained (Figures 2A, B). After the intersection, a total of 161 common DEGs were obtained (Figure 2C; Table 2).

**TABLE 2:** 161 common DEGs of GSE 55235 and GSE 55457.

Regulation	161 common DEGs									
**Up (73)**	CX3CR1	LTC4S	WNT5B	ANOS1	TNFSF11	LRRC17	SCRG1	EPYC	MGAT4C	TLR7
	C1QTNF3	COPZ2	TREM2	NAP1L3	LTA4H	TRIL	SLC5A3	SLC18A2	GUCA1A	LRRC15
	TDO2	OGN	RTN1	LIPC	PNMA8A	ANKH	CAPG	RTP4	PTN	MTUS2
	PTHLH	CBR3	PTGS1	POU2AF1	NELL1	GPR1	DPT	HTR2B	DPYS	TMEM106C
	ZNF668	GPM6A	RPE65	TAC1	RGS13	NUDT11	FANCF	LCK	ZKSCAN4	MYOM2
	FGGY	GPR88	NDUFA4L2	ST8SIA1	RRAS	TNFRSF11B	MSTN	NUDT1	SIL1	TNIP3
	GLRB	STMN2	GSTZ1	CACNA2D3	ERMAP	WIF1	CLIC3	ERAP2	HSD11B2	MS4A1
	PDZRN4	APOC4	ZIC1							
**Down (88)**	SLC19A2	FOSL2	SIK1	NFIL3	KLF4	MAFF	GADD45B	MYC	TIPARP	APOLD1
	ZFP36	PPP1R15A	DUSP2	CDKN1A	ATF3	SOD2	BTG2	CXCL2	ADAMTS1	CCNL1
	SLC16A7	TNFAIP3	CCN1	NEDD9	ETS2	NAMPT	FOSB	SLC2A3	GRB10	NPAS2
	VEGFA	DUSP5	NR4A2	PTGS2	ZFP36L2	CXCL3	CRISPLD2	EDNRB	KLF6	STC1
	JUN	RND1	EGR1	IL1R1	JUNB	INHBB	SPRY1	HAS1	SLC2A14	FOXO3B
	IRAK3	DUSP1	SOCS3	LAMA3	NR4A1	IL6	RGS16	FKBP5	VEGFD	PFKFB3
	PHACTR1	LRCH1	ZMYM2	RGS1	ARNTL	FOXC2	SLC7A5	EFNB2	NR4A3	MCF2L
	RPS4Y1	SNAI1	CCL20	LGALSL	DDX3Y	ACACB	THBD	CCL25	DUSP4	KDM5D
	SELE	NAA15	FOSL1	USP9Y	SBNO2	KLF13	ANKRD11	NLGN4Y		

### 3.2 Enrichment analysis and protein-protein interaction analysis of DEGs in OA

**FIGURE 3:**
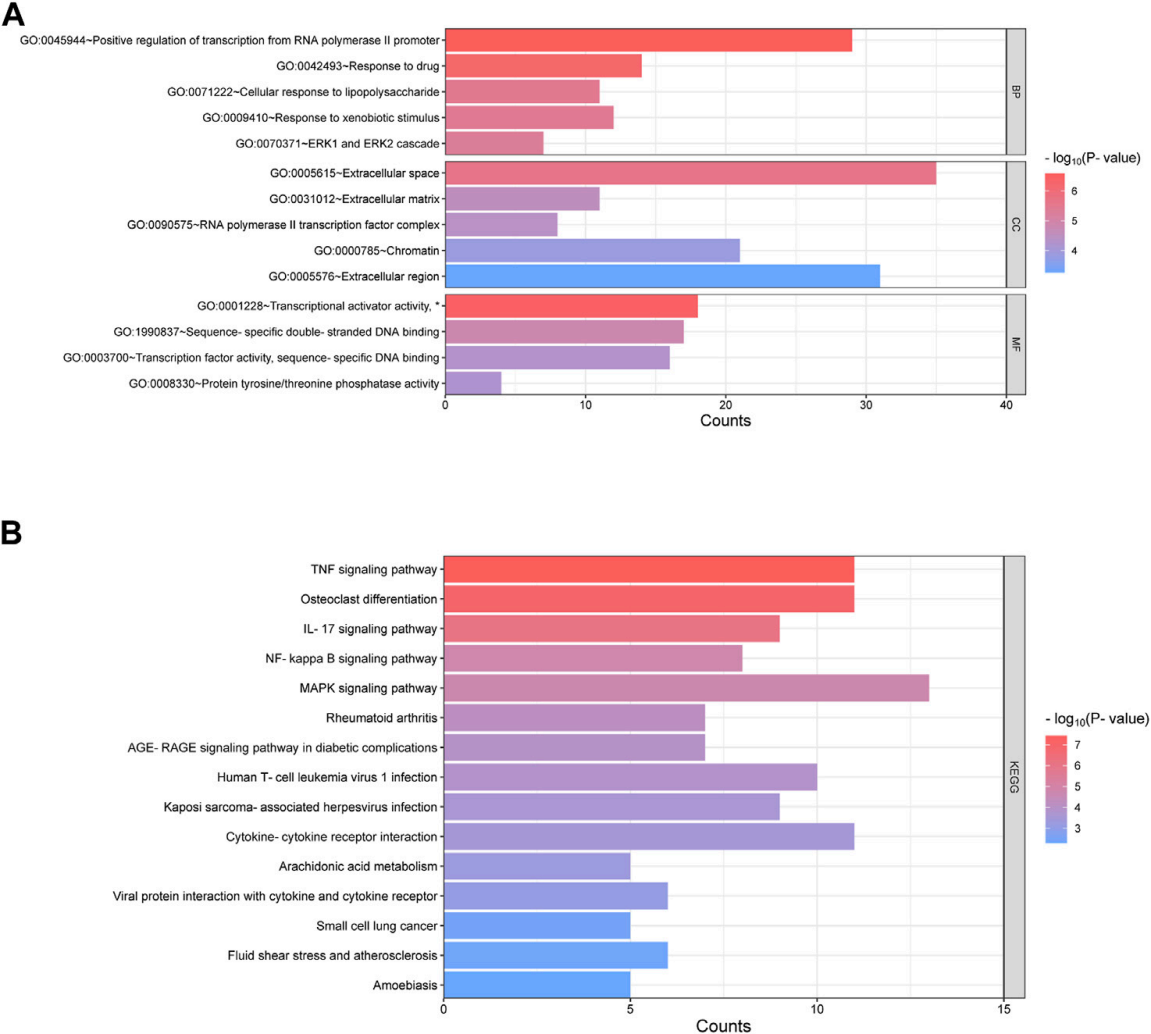
Enrichment of GO and KEGG of 161 common DEGs. **(A)** Go annotation categories of 161 common DEGs including top 5 of BP, top 5 of CC and top 4 of MF. The horizontal axis represents counts of enriched genes. **(B)** Top 15 KEGG pathway categories after enrichment analysis of 161 common DEGs. The horizontal axis represents counts of enriched genes. GO = gene ontology; BP = biological processes; CC = cellular components; MF = molecular function; KEGG = Kyoto Encyclopedia of Genes and Genomes. (*represents “RNA polymerase II transcription regulatory region sequence-specific binding”, which was hided due to the space).

**FIGURE 4:**
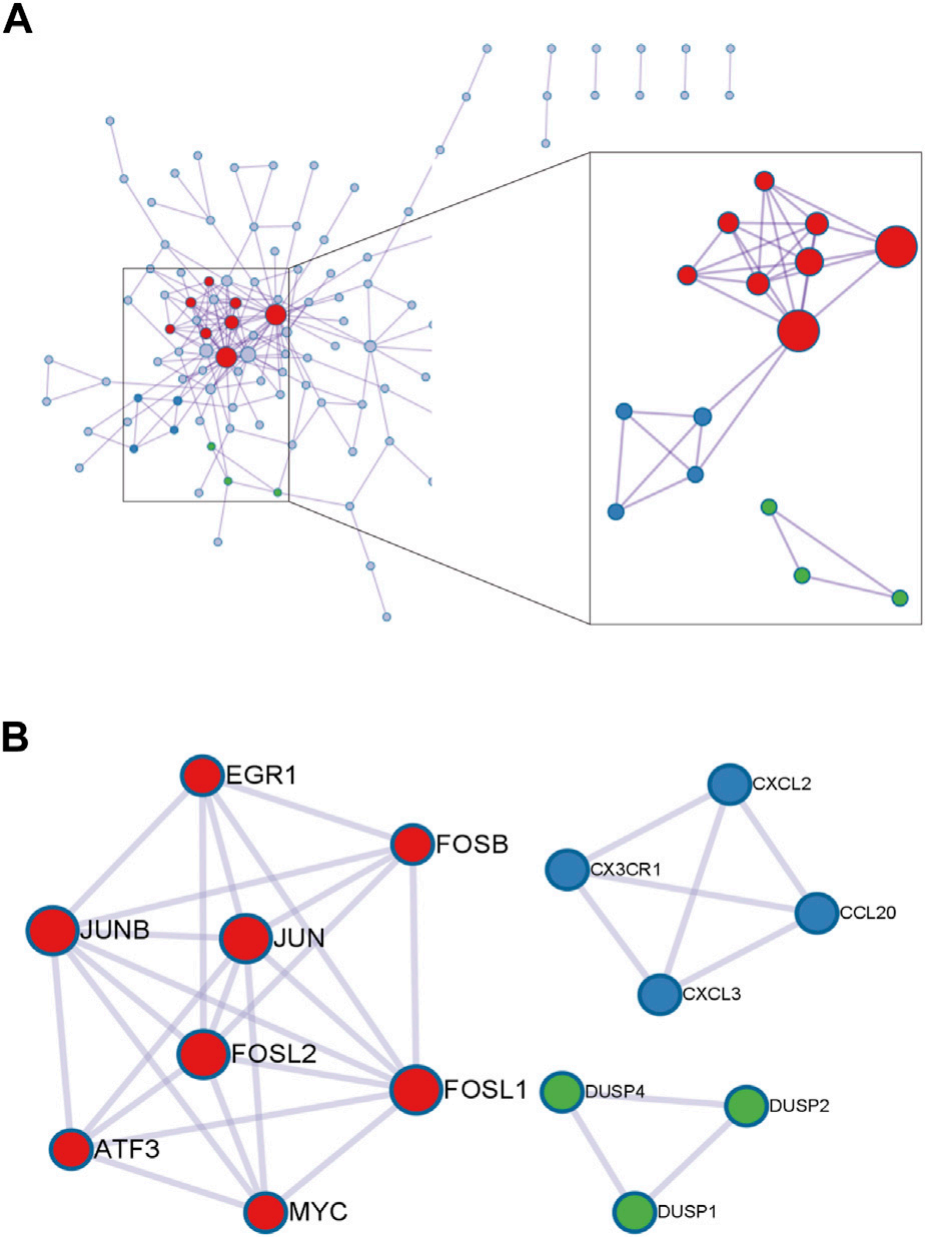
PPI analysis of 161 DEGs and screening of key modules. **(A)** Overview of the PPI network of 161 DEGs through metascape. The larger size of the points, the higher degree of the genes. **(B)** Genes in three key modules with the highest MCODE value.

In order to further explore the function of DEGs in OA, GO annotation and KEGG enrichment analysis were performed subsequently. As the results, 156 GO terms with *p* < 0.05 were screened out (S1). Top 5 BP, 5 CC and 4 MF terms were showed in Figure 3A. It showed that the most significant BP of 161 commom DEGs was “positive regulation of transcription from RNA polymerase II promoter”, the most significant CC was “extracellular space”, and the most significant MF was “transcriptional activator activity, RNA polymerase II transcription regulatory region sequence-specific binding”. As the result of KEGG enrichment analysis, 38 pathways with *p* < 0.05 were found (S2), and top 15 pathways were showed in Figure 3B. It showed that these genes were involved in pathways including tumor necrosis factor (TNF) signaling pathway, osteoclast differentiation, IL-17 signaling pathway, nuclear factor kappa-B (NF-κB) signaling pathway and so on. In addition, as the result of PPI analysis combined with MCODE component algorithm, top 3 modules with highest MCODE score were showed in Figures 4A, B. Finally, 8 hub genes in the biggest module were identified as hub genes, which were ATF3, EGR1, FOSB, FOSL1, FOSL2, JUN, JUNB, and MYC (Table 3).

**TABLE 3:** Features and functions of 8 hub genes in OA screened from DEGs.

Gene	Full name	Function	Regulation in OA synovium
ATF3	Activating transcription factor 3	ATF3 suppresses cyclin D1 expression in chondrocytes James et al. (2006)	Down
EGR1	Early growth response 1	EGR1 in chondrocytes could accelerate chondrocyte hypertrophy, prevent COl2A1	Down
		expression, and promote the release of inflammatory factors Sun et al. (2019)	
FOSB	FBJ murine osteosarcoma viral	FOSB promotes cell proliferation and inhibit apoptosis as a kind of proto	Down
	oncogene homolog B	oncogene Papoudou-Bai et al. (2017)	
FOSL1	FOS-like antigen 1	FOSL1 plays an oncogenic role by modulating various cellular processes Dhillon and Tulchinsky (2015)	Down
FOSL2	FOS-like antigen 2	The leucine chain encoded by FOSL2 dimerizes with the protein encoded by JUN	Down
		family to synthesize into AP1 Guo et al. (2019)	
JUN	Jun proto-oncogene	JUN family includes v-Jun, c-Jun, Jun B and Jun D.c-Jun transactivates Puma gene	Down
		expression to promote OA Lu et al. (2014)	
JUNB	Jun B proto-oncogene	Jun B positively regulates the expression of IL-4 in Th2 cells and regulates MMP13	Down
		expression Licht et al. (2006); Tanel et al. (2009)	
MYC	MYC proto-oncogene	MYC encodes transcription factors to regulate transcriptional activity and cell	Down
		proliferation, growth, and apoptosis O'Donnell et al. (2005)	

### 3.3 Correlation analysis between OA hub genes and ferroptosis related genes

**FIGURE 5:**
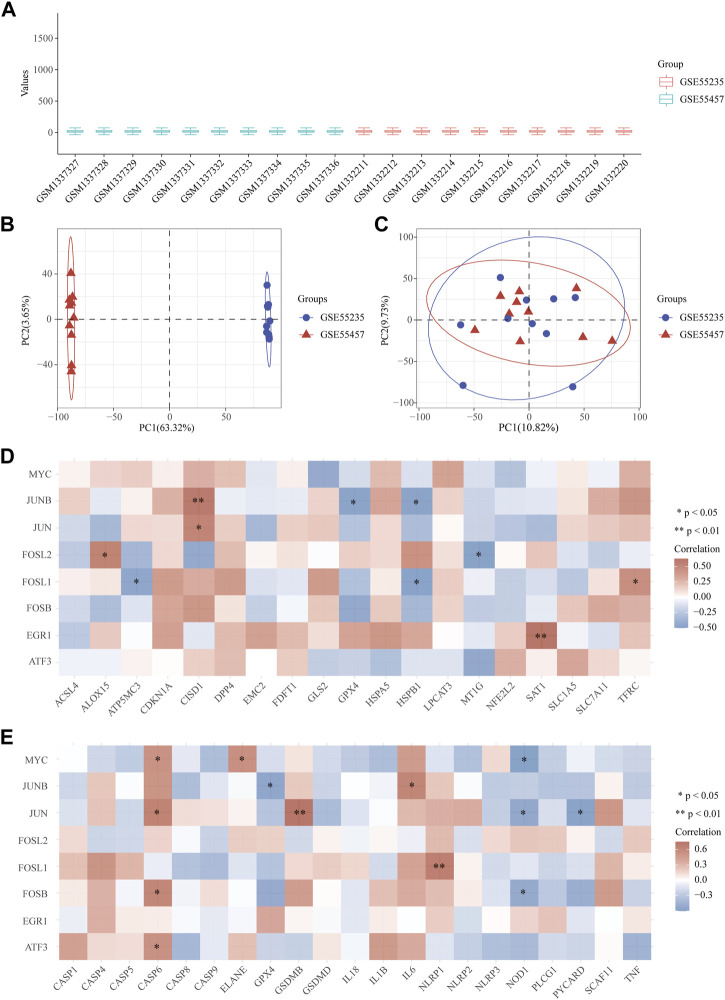
Correlation between OA hub genes and ferroptosis or pyroptosis related genes. **(A)** The data standardization evaluated by box diagram after the combination and normalization of 2 groups. **(B, C)** The visual PCA diagram before **(B)** and after **(C)** removal of the batch. **(D, E)** The heat map of the correlation between hub genes and genes related to ferroptosis and pyroptosis. The horizontal and vertical coordinates represent genes. Different colors represent the correlation coefficient (red represents positive correlation, blue represents negative correlation). PC = Principal Component.

We combined and normalized the data of 2 groups as described above, then evaluated the data standardization and batch effect through the box diagram (Figure 5A) and visual PCA diagram (Figure 5B), respectively. 19 ferroptosis related genes and 21 pyroptosis related genes were selected for correlation analysis and displayed by heat map. It showed that in the analysis of ferroptosis, ALOX15, CISD1, SAT1, and TFRC were positively correlated to hub genes, while ATP5MC3, GPX4, HSPB1, and MT1G were negatively correlated (Figure 5C). In the analysis of pyroptosis, Caspase-6, ELANE, GSDMB, IL-6 and NLRP1 were positively correlated to hub genes, while GPX4, NOD1 and PYCARD were negatively correlated (Figure 5D).

### 3.4 Analysis of ceRNA regulatory network of OA hub genes

**FIGURE 6:**
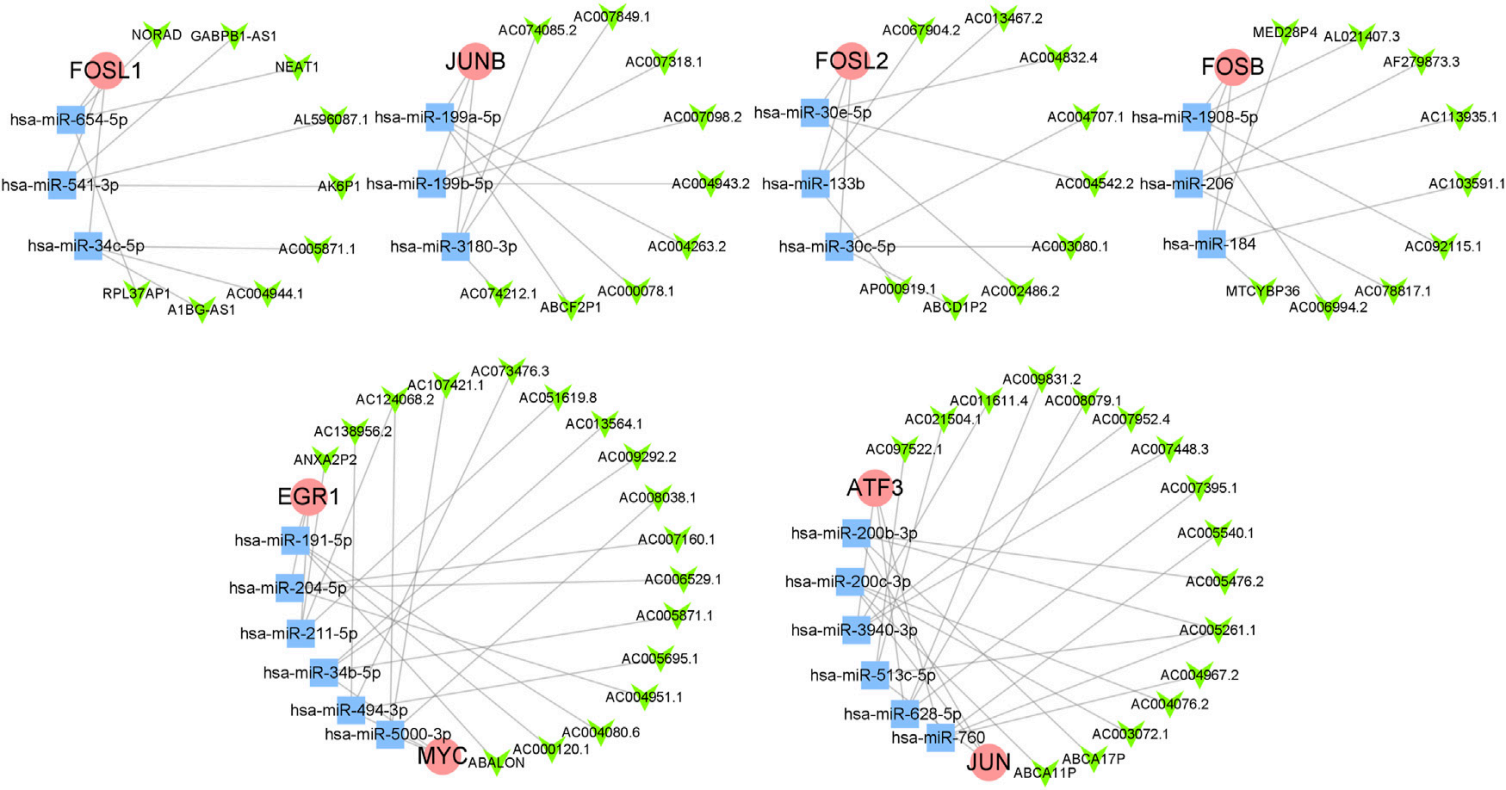
The ceRNA regulatory network of hub genes. The red circles represent hub genes, the blue squares represent miRNAs that targeted hub genes, and the green V-shapes represent lncRNAs that targeted miRNAs.

A total of 24 reliable miRNAs that regulated mRNA of 8 hub genes were obtained, after the intersection of the prediction, using ENCORI and TargetScan database. Then, a total of 69 reliable lncRNAs were obtained after the analysis under LncBase v3.0 database and selection, which regulated miRNAs above. Finally, we constructed the ceRNA regulatory network of hub genes (Figure 6).

### 3.5 Validation of hub genes

**FIGURE 7:**
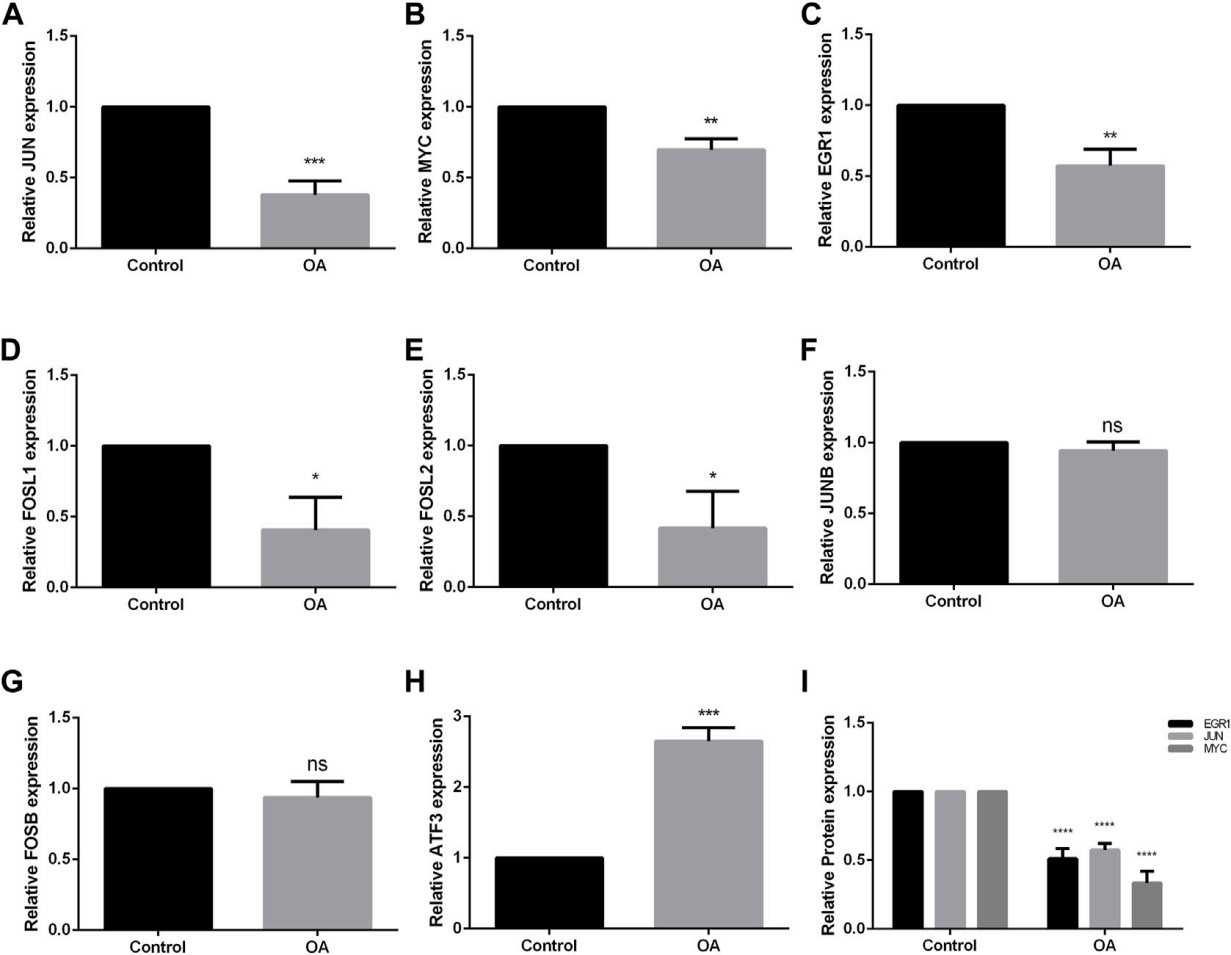
Validation of hub genes through qRT-PCR and ELISA of FLS. **(A–H)** mRNA expression of JUN, MYC, EGR1, FOSL2, FOSL1, JUNB, FOSB and ATF3. JUN, MYC, EGR1, FOSL2 and FOSL1 showed the same trend as the results of bioinformatics analysis. JUNB and FOSB showed no significant difference between two groups, while MYC showed the opposite trend. **(I)** The result of ELISA analysis of EGR1, JUN and MYC in the supernatant of FLS. OA = osteoarthritis. ^*^ *p* < 0.05, ^**^ *p* < 0.01, ^***^ *p* < 0.001, ^****^ *p* < 0.0001. Error bars represent SD.

qRT-PCR and ELISA were used to evaluate the mRNA and protein expression level of hub genes in FLS of control group and OA group. The results showed that the mRNA expression levels of JUN, MYC, EGR1, FOSL1, and FOSL2 of FLS in OA group was significantly lower than that in normal FLS (*p* < 0.001, *p* < 0.01, *p* < 0.01, *p* < 0.05, *p* < 0.05, respectively), meeting the trend of bioinformatics analysis (Figures 7A–E). The expression of JUNB and FOSB had no significant difference with control group (Figures 7F, G), while the expression of ATF3 was higher than that in normal FLS (*p* < 0.001, Figure 7H), which was opposite to bioinformatics analysis. The result of ELISA showed that the expression of EGR1, JUN, MYC were all significantly lower in the supernatant of FLS in OA group, same to the result of qRT-PCR (*p* < 0.0001, Figure 7I).

### 3.6 Identification of potential drugs for OA

**FIGURE 8:**
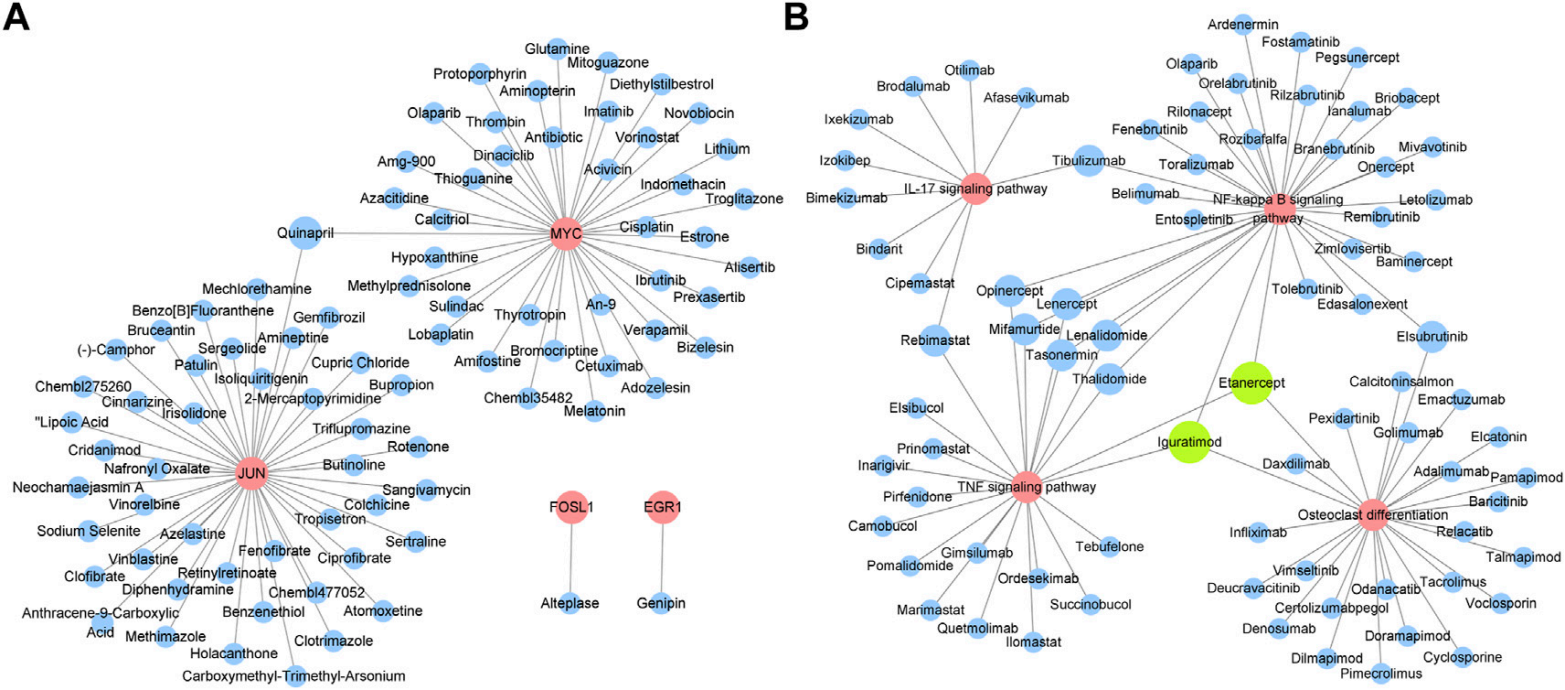
Identification of potential drugs for OA. **(A)** Drugs targeting hub genes. Red circles represent genes, blue circles represent drugs. **(B)** Drugs targeting top four KEGG pathways. Red circles represent pathways, blue circles represent drugs. Green circles represent drugs which have three targets.

Drugs targeting 8 hub genes and top 4 KEGG pathways were identified by CTD, DrugBank and DGI database. As the result, 85 drugs targeting 4 of hub genes were obtained, among which Quinapril targeted MYC and JUN at the same time (Figure 8A). 285 drugs were found targeting KEGG pathways, among which Elsubrutinib, Lenalidomide, Lenercept, Mifamurtide, Opinercept, Rebimastat, Tasonermin, Thalidomide and Tibulizumab targeted two pathways. Etanercept and Iguratimod targeted three pathways, which were selected for following validation (Table 4; Figure 8B).

**TABLE 4:** Drugs targeting KEGG pathways ≥2.

Drug	Target pathways	Functions or applications
Etanercept	Osteoclast differentiation	Fusion protein that binds TNF-α
	NF-kappa B signaling pathway	Treatment for severe rheumatoid arthritis
	TNF signaling pathway	Treatment for moderate to severe plaque psoriasis
Iguratimod	Osteoclast differentiation	Under investigation in rheumatoid arthritis
	NF-kappa B signaling pathway	
	TNF signaling pathway	
Elsubrutinib	Osteoclast differentiation	Bruton’s tyrosine kinase inhibitor
	NF-kappa B signaling pathway	
Lenalidomide	NF-kappa B signaling pathway	Anti multiple myeloma and anemia
	TNF signaling pathway	
Lenercept	NF-kappa B signaling pathway	TNF receptor fusion protein
	TNF signaling pathway	Under investigation in multiple sclerosis
Mifamurtide	NF-kappa B signaling pathway	Antineoplasitc
	TNF signaling pathway	
Opinercept	NF-kappa B signaling pathway	TNF-α inhibitor
	TNF signaling pathway	Under investigation in rheumatoid arthritis
Rebimastat	IL-17 signaling pathway	Under investigation treatment in lung cancer and prostate cancer
	TNF signaling pathway	
Tasonermin	NF-kappa B signaling pathway	Recombinant soluble form of TNF-α
	TNF signaling pathway	An adjunt to surgery to remove soft tissue sarcomas of the limbs
Thalidomide	NF-kappa B signaling pathway	Antineoplasitc
	TNF signaling pathway	
Tibulizumab	IL-17 signaling pathway	Bispecific antibody targeting BAFF and IL-17A
	NF-kappa B signaling pathway	

### 3.7 Etanercept and Iguratimod protect FLS of OA from inflammation and cartilage degeneration

**FIGURE 9:**
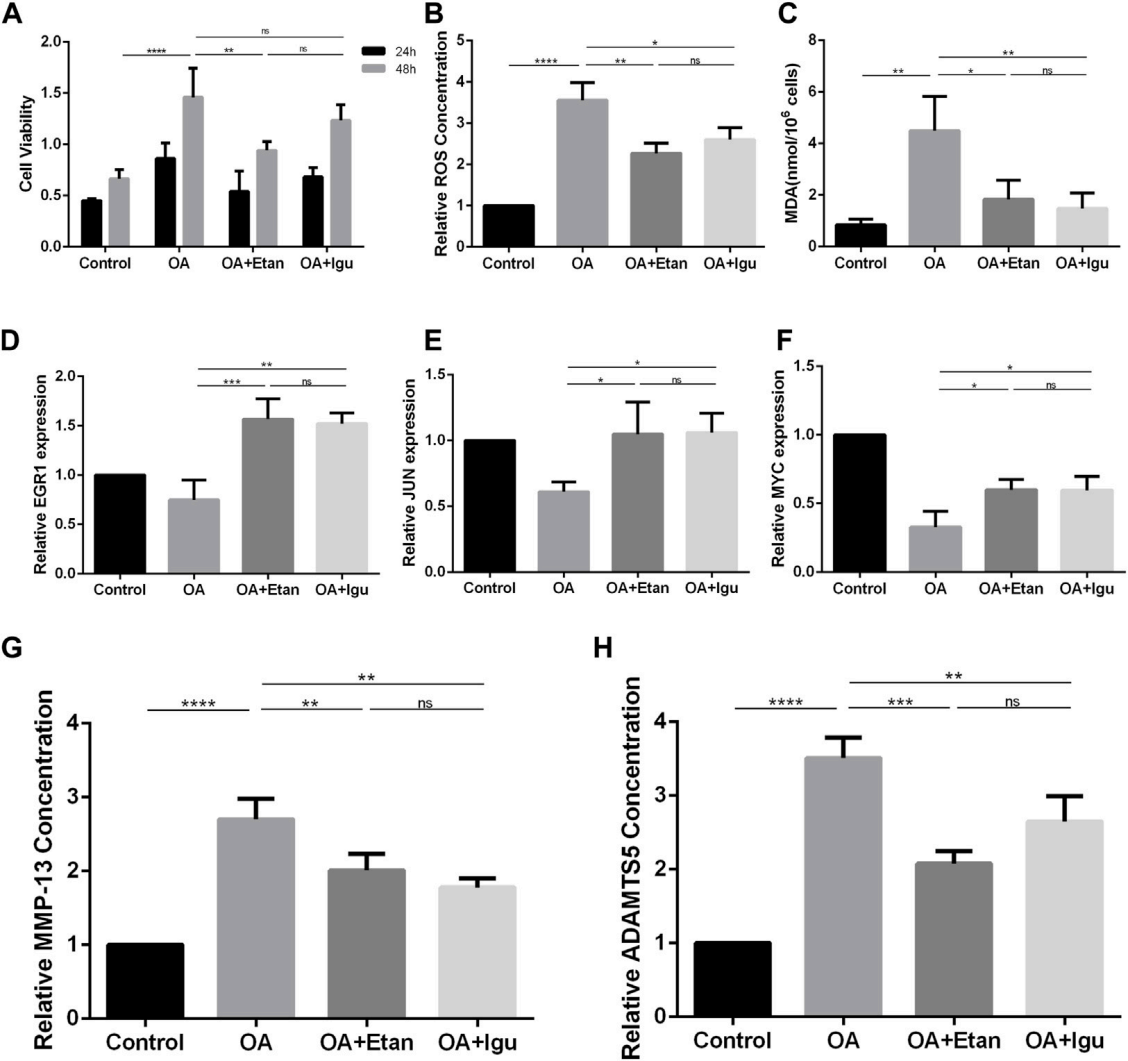
Validation of the effect of Etanercept and Iguratimod on FLS. **(A)** Cell viability of FLS at 24 and 48 h. **(B)** Relative Intracellular ROS concentration of FLS. **(C)** MDA concentration of FLS of different groups. **(D–F)** mRNA expression of EGR1, JUN and MYC of the four groups. **(G, H)** The level of MMP-13 and ADAMTS5 in the supernatant of FLS in different groups. OA = osteoarthritis; Etan = Etanercept; Igu = Iguratimod; ROS = reactive oxygen species; MDA = Malonic dialdehyde; MMP-13 = matrix metalloproteinase 13; ADAMTS5 = a disintegrin and metalloproteinase with thrombospondin motifs-5. ^*^ *p* < 0.05, ^**^ *p* < 0.01, ^***^ *p* < 0.001, ^****^ *p* < 0.0001. Error bars represent SD.

To verify the effect of Etanercept and Iguratimod on FLS, we co-treated FLS with IL-1β. By the test of cell viability, we found that Etanercept can significantly inhibit the growth rate of FLS compared with OA group (*p* < 0.01, Figure 9A). The results of intracellular ROS and MDA detection showed that the levels of these two species in Etanercept and Iguratimod groups were significantly lower than those of OA group (*p* < 0.01, *p* < 0.05, Figures 9B, C). At the same time, the mRNA expression of EGR1, JUN and MYC in Etanercept and Iguratimod group were significantly higher than those in OA group (*p* < 0.001, *p* < 0.01 for EGR1, *p* < 0.001, *p* < 0.05 for JUN and MYC, Figures 9D–F). Moreover, the level of MMP-13 and ADAMTS5 in the supernatant of FLS was significantly lower in Etanercept and Iguratimod group than those in OA group (*p* < 0.01 for MMP-13, *p* < 0.001, *p* < 0.01 for ADAMTS5, Figures 9G, H).

## 4 Discussion

As one of the major chronic diseases endangering the middle-aged and elderly, the affected population of OA is also showing a trend of younger age (Mahmoudian et al., 2021). The compliance of non-drug treatment such as kinesitherapy and physiotherapy is usually inexact. The operations for KOA mainly includes arthroscopic debridement, high tibial osteotomy (HTO), unicondylar knee arthroplasty (UKA) and TKA (Zhang et al., 2020), which still have the problems of high risk and cost. The gathered evidence suggests that mononuclear infiltration and over expression of inflammatory mediators in synovium are seen in early OA and predate radiographic damage in OA (Sokolove and Lepus, 2013). Synovium and synovial fluid are the main contributors to inflammation that can secrete key cytokines, most of which are the main regulators of matrix metalloproteinases (MMP), resulting in the loss of cartilage (Kulkarni et al., 2021). Therefore, in-depth study of the molecular mechanism of synovitis is of great significance to the prevention and treatment for OA.

In this study, we screened out 8 hub genes in synovim that may be involved in the progression of OA through a series of bioinformatics methods. EGR1 is potentially involved in postnatal bone biology and implicated in the regulation of osteoblastic cell growth and differentiation. Studies have shown that when EGR1 was inhibited, the bone mass of the limbs decreased in mice, with lower bone volume fraction and mineral density, same to our study (Reumann et al., 2011). However, over-expression of EGR1 regulated the expression of KLF5 and β-catenin signaling pathway, leading to the acceleration of cartilage hypertrophy and degeneration (Sun et al., 2019). MYC encodes transcription factors to regulate transcriptional activity and cell proliferation, growth, and had also been found to regulate OA process through multiple pathways. Wu et al. (O'Donnell et al., 2005) found that the effects of miR-24 on OA chondrocytes may be achieved by targeting MYC and further regulating the MAPK signaling pathway. The silence of c-MYC could promote proliferation of rat chondrocyte (Zou et al., 2018). ATF3 deficiency in chondrocytes had been reported to alleviates OA development (Iezaki et al., 2016). This effect may be achieved by ATF3 directly affecting the expression of MMP-13 thus reducing cartilage loss (Chan et al., 2017). However, There is little study on the mechanism of ATF3 in OA synoviocyte, except for the bioinformatics analysis.

The rest five hub genes, which were FOSB, FOSL1, FOSL2, JUN and JUNB, belonged to the AP-1 family. AP-1 was thought to be critically involved in the pathogenesis of arthritis due to the binding activity for its cognate recognition sites in the promoters of inflammatory cytokines and matrix-degrading enzymes (Huber et al., 2019). Study showed that mitochondrial dysfunction triggered a catabolic response in chondrocytes *via* activation of the JNK/AP1 pathway (Ansari et al., 2020). On the other hand, JUNB-FBXO21-ERK axis promoted cartilage degeneration in OA by inhibiting autophagy (Lin et al., 2021).

As independent forms of programmed death, ferroptosis and pyroptosis have been shown to play essential roles in the pathological processes of tumor, Alzheimer’s disease, cerebral hemorrhage, ischemia-reperfusion injury, OA and so on (Kenny et al., 2019; Liang et al., 2019; Liu et al., 2020b). In this study, we selected several ferroptosis and pyroptosis related genes. Although these genes were not differentially expressed in the two datasets, analyzing their relationship with hub genes will be still beneficial to explore the mechanism of FLS participating in OA. We found that FOSL1 and JUNB were associated with 3 of the 19 ferroptosis related genes. Overexpression of c-JUN inhibits ferroptosis induced by erastin in Schwann cells to promotes the rehabilitation of facial nerve function (Gao et al., 2022). Vertically, the strongest two genes related to hub genes were CISD1 and HSPB1. CISD1, an iron-containing outer mitochondrial membrane protein, inhibits ferroptosis by protection against mitochondrial lipid peroxidation (Yuan et al., 2016). The phosphorylation of HSPB1 at the Ser-15 site induced by Erastin is the key of the protective response to ferroptosis stress (Wang et al., 2022). In addition, over-expression of HSPB1 attenuates ferroptosis in rats through promoting G6PD expression (Dai and Hu, 2022). Therefore, we believe that CISD1 and HSPB1 are worth studying in the future to explore the mechanism of ferroptosis in OA synovium. In addition, we found that JUN was related to the most pyroptosis related genes. Study had showed that the Jun N-terminal kinases (JNK) pathway is largely upstream of the NLRP3 inflammasome, which exerts a crucial regulatory impact on microglia pyroptosis and inflammatory responses (He et al., 2021). However, the relationship between JUN and pyroptosis of FLS still needs further study. As a key regulator of innate immunity and inflammasome activation, caspase-6 promotes the activation of pyroptosis (Zheng et al., 2020).

Studies have found the significance of miRNAs and lncRNAs on OA. Xu et al. (2021) showed that SNHG7 ameliorated the development of OA by suppressing apoptosis through miR-214-5p-PPARGC1B-PPARγ axis. Wang et al. (2019) found that lncRNA FOXD2 Adjacent Opposite Strand RNA 1 (FOXD2-AS1) served as the protector for OA patients by inducing chondrocyte proliferation. The downregulation of lncRNA LOC101928134, which acts as a promoter of OA, can block the process of OA (Yang et al., 2019). Here, we built a ceRNA network after predicting the upstream miRNAs and lncRNAs, in order to provide more possible early biomarkers for diagnosis, or targeting drugs for OA patients.

Among the 11 drugs targeting two or more than two KEGG pathways, 10 acted on NF-κB signaling pathway, 9 acted on TNF signaling pathway, 3 acted on Osteoclast differentiation pathway, and 2 acted on IL-17 signaling pathway, suggesting that NF-κB was still the key point for OA treatment. Moreover, Etanercept and Iguratimod related to three pathways. Etanercept, a soluble fusion protein that binds TNF-α, has been proved to be an effective choice targeting several inflammatory diseases, especially rheumatoid arthritis (Graudal et al., 2015). Recent study showed that Etanercept lead to the repairment of cartilage with a special scaffold (Campos et al., 2022), and the reduction of MMP-3 (Kroon et al., 2020), suggesting the potential effect on OA. The effect of Iguratimod on OA still needs further study. Our study proved that both Etanercept and Iguratimod could reduce level of intracellular ROS and MDA of FLS as well as level of MMP-13 and ADAMTS5 in the supernatant. In addition, Etanercept could significantly alleviate the abnormal proliferation of FLS under OA environment. Drugs targeted to the hub genes can be divided into antineoplastic, antipsychotic, anti-inflammatory, anti-hyperglycemia, anti-hyperlipidemia, etc. Their roles in OA still needs to be explored.

Our study still had some limitations. First, we obtained gene arrays in synovial tissues of OA patients from GEO database. However, due to the lack of information of patients, it was hard to correlate the DEGs and hub genes obtained in this study with the severity or grade of OA. Second, the sample size of each group was not so large, which was needed for further research.

In summary, our study aimed to identify key genes involved in the pathophysiology of OA. 161 common DEGs and 8 hub genes were screened through GO analysis, KEGG pathway analysis, PPI network construction as well as MCODE, which may become potential targeting clinical diagnosis and treatment of OA. Furthermore, 5 of 8 genes met the similar expression trend with our result through the validation. Subsequently, we discussed the relationship between hub genes and key genes of ferroptosis and pyroptosis. Moreover, miRNAs and lncRNAs were identified to construct the ceRNA regulatory network of hub genes. Finally, we found that Etanercept and Iguratimod, as top two of potential drugs targeting KEGG pathways, had the protective effect on FLS in the OA environment.

## 5 Conclusion

EGR1, JUN, MYC, FOSL1, and FOSL2 were identified and validated as hub genes in the development of OA after series of bioinformatics analysis. They may have effect on OA development through different kinds of pathways, including the process of ferroptosis and pyroptosis. Etanercept and Iguratimod seemed to have stronger opportunities to be novel drugs for OA.

## Data Availability

The datasets presented in this study can be found in online repositories. The names of the repository/repositories and accession number(s) can be found in the article/Supplementary Material.
