# Supplementary material for: Identification and validation of hub genes and potential drugs involved in osteoarthritis through bioinformatics analysis
Source: Front Genet. 2023 Feb 9;14:1117713. doi: 10.3389/fgene.2023.1117713 (PMC9947480; doi:10.3389/fgene.2023.1117713)
Supplement: Supplementary file 1 [file Table1.DOCX]

**Supplementary Table 1. GO Terms (BP, CC and MF) of 161 common DEGs with *P* < 0.05**

| **Term** | **Count** | **%** | **P-Value** |
| --- | --- | --- | --- |
| GO:0045944~positive regulation of transcription from RNA polymerase II promoter | 29 | 17.06 | 2.61E-07 |
| GO:0001228~transcriptional activator activity, RNA polymerase II transcription regulatory region sequence-specific binding | 18 | 10.59 | 4.17E-07 |
| GO:0042493~response to drug | 14 | 8.24 | 6.15E-07 |
| GO:0005615~extracellular space | 35 | 20.59 | 2.32E-06 |
| GO:0071222~cellular response to lipopolysaccharide | 11 | 6.47 | 3.25E-06 |
| GO:0009410~response to xenobiotic stimulus | 12 | 7.06 | 3.69E-06 |
| GO:0070371~ERK1 and ERK2 cascade | 7 | 4.12 | 4.69E-06 |
| GO:0006954~inflammatory response | 15 | 8.82 | 5.35E-06 |
| GO:1990837~sequence-specific double-stranded DNA binding | 17 | 10.00 | 1.41E-05 |
| GO:0031012~extracellular matrix | 11 | 6.47 | 2.87E-05 |
| GO:0045444~fat cell differentiation | 7 | 4.12 | 3.32E-05 |
| GO:0090575~RNA polymerase II transcription factor complex | 8 | 4.71 | 3.90E-05 |
| GO:0003700~transcription factor activity, sequence-specific DNA binding | 16 | 9.41 | 5.32E-05 |
| GO:0008330~protein tyrosine/threonine phosphatase activity | 4 | 2.35 | 6.22E-05 |
| GO:0008285~negative regulation of cell proliferation | 14 | 8.24 | 8.42E-05 |
| GO:0005515~protein binding | 126 | 74.12 | 8.55E-05 |
| GO:0008201~heparin binding | 9 | 5.29 | 1.04E-04 |
| GO:0007565~female pregnancy | 7 | 4.12 | 1.15E-04 |
| GO:0001706~endoderm formation | 4 | 2.35 | 1.31E-04 |
| GO:0000785~chromatin | 21 | 12.35 | 1.40E-04 |
| GO:0017017~MAP kinase tyrosine/serine/threonine phosphatase activity | 4 | 2.35 | 1.45E-04 |
| GO:0035259~glucocorticoid receptor binding | 4 | 2.35 | 2.29E-04 |
| GO:0032496~response to lipopolysaccharide | 8 | 4.71 | 2.67E-04 |
| GO:0000978~RNA polymerase II core promoter proximal region sequence-specific DNA binding | 23 | 13.53 | 3.47E-04 |
| GO:0008217~regulation of blood pressure | 6 | 3.53 | 4.34E-04 |
| GO:0006935~chemotaxis | 7 | 4.12 | 4.42E-04 |
| GO:0000188~inactivation of MAPK activity | 4 | 2.35 | 5.02E-04 |
| GO:0051591~response to cAMP | 5 | 2.94 | 5.16E-04 |
| GO:0005576~extracellular region | 31 | 18.24 | 5.51E-04 |
| GO:0035914~skeletal muscle cell differentiation | 5 | 2.94 | 5.60E-04 |
| GO:0071376~cellular response to corticotropin-releasing hormone stimulus | 3 | 1.76 | 6.13E-04 |
| GO:0000122~negative regulation of transcription from RNA polymerase II promoter | 19 | 11.18 | 6.99E-04 |
| GO:1902895~positive regulation of pri-miRNA transcription from RNA polymerase II promoter | 5 | 2.94 | 7.60E-04 |
| GO:0030282~bone mineralization | 5 | 2.94 | 8.77E-04 |
| GO:0035970~peptidyl-threonine dephosphorylation | 4 | 2.35 | 9.84E-04 |
| GO:0006357~regulation of transcription from RNA polymerase II promoter | 27 | 15.88 | 0.0010 |
| GO:0005125~cytokine activity | 8 | 4.71 | 0.0010 |
| GO:0043065~positive regulation of apoptotic process | 10 | 5.88 | 0.0013 |
| GO:0007612~learning | 5 | 2.94 | 0.0015 |
| GO:0008284~positive regulation of cell proliferation | 13 | 7.65 | 0.0015 |
| GO:0046697~decidualization | 4 | 2.35 | 0.0015 |
| GO:0042802~identical protein binding | 27 | 15.88 | 0.0017 |
| GO:0051602~response to electrical stimulus | 4 | 2.35 | 0.0019 |
| GO:0005667~transcription factor complex | 8 | 4.71 | 0.0020 |
| GO:0060325~face morphogenesis | 4 | 2.35 | 0.0020 |
| GO:0051019~mitogen-activated protein kinase binding | 4 | 2.35 | 0.0021 |
| GO:0044344~cellular response to fibroblast growth factor stimulus | 4 | 2.35 | 0.0022 |
| GO:0070098~chemokine-mediated signaling pathway | 5 | 2.94 | 0.0023 |
| GO:0009611~response to wounding | 5 | 2.94 | 0.0023 |
| GO:0048661~positive regulation of smooth muscle cell proliferation | 5 | 2.94 | 0.0023 |
| GO:0031640~killing of cells of other organism | 5 | 2.94 | 0.0024 |
| GO:0008083~growth factor activity | 7 | 4.12 | 0.0026 |
| GO:0003677~DNA binding | 22 | 12.94 | 0.0030 |
| GO:0001938~positive regulation of endothelial cell proliferation | 5 | 2.94 | 0.0033 |
| GO:0006366~transcription from RNA polymerase II promoter | 8 | 4.71 | 0.0033 |
| GO:0035335~peptidyl-tyrosine dephosphorylation | 4 | 2.35 | 0.0033 |
| GO:0001666~response to hypoxia | 7 | 4.12 | 0.0034 |
| GO:0000981~RNA polymerase II transcription factor activity, sequence-specific DNA binding | 21 | 12.35 | 0.0041 |
| GO:0030316~osteoclast differentiation | 4 | 2.35 | 0.0042 |
| GO:0043409~negative regulation of MAPK cascade | 4 | 2.35 | 0.0048 |
| GO:0016702~oxidoreductase activity, acting on single donors with incorporation of molecular oxygen, incorporation of two atoms of oxygen | 3 | 1.76 | 0.0049 |
| GO:0045893~positive regulation of transcription, DNA-templated | 14 | 8.24 | 0.0050 |
| GO:0070373~negative regulation of ERK1 and ERK2 cascade | 5 | 2.94 | 0.0053 |
| GO:0002548~monocyte chemotaxis | 4 | 2.35 | 0.0054 |
| GO:0071456~cellular response to hypoxia | 6 | 3.53 | 0.0056 |
| GO:0043565~sequence-specific DNA binding | 9 | 5.29 | 0.0059 |
| GO:0019722~calcium-mediated signaling | 5 | 2.94 | 0.0061 |
| GO:0004721~phosphoprotein phosphatase activity | 4 | 2.35 | 0.0063 |
| GO:0071277~cellular response to calcium ion | 5 | 2.94 | 0.0064 |
| GO:0005161~platelet-derived growth factor receptor binding | 3 | 1.76 | 0.0065 |
| GO:0051781~positive regulation of cell division | 4 | 2.35 | 0.0069 |
| GO:0045779~negative regulation of bone resorption | 3 | 1.76 | 0.0078 |
| GO:0008009~chemokine activity | 4 | 2.35 | 0.0080 |
| GO:0030154~cell differentiation | 13 | 7.65 | 0.0081 |
| GO:0042542~response to hydrogen peroxide | 4 | 2.35 | 0.0085 |
| GO:0010507~negative regulation of autophagy | 4 | 2.35 | 0.0094 |
| GO:0043066~negative regulation of apoptotic process | 11 | 6.47 | 0.0097 |
| GO:0031668~cellular response to extracellular stimulus | 3 | 1.76 | 0.0097 |
| GO:0005634~nucleus | 60 | 35.29 | 0.0106 |
| GO:0055093~response to hyperoxia | 3 | 1.76 | 0.0108 |
| GO:0044849~estrous cycle | 3 | 1.76 | 0.0108 |
| GO:0048146~positive regulation of fibroblast proliferation | 4 | 2.35 | 0.0109 |
| GO:0061844~antimicrobial humoral immune response mediated by antimicrobial peptide | 5 | 2.94 | 0.0110 |
| GO:0002042~cell migration involved in sprouting angiogenesis | 3 | 1.76 | 0.0118 |
| GO:0051412~response to corticosterone | 3 | 1.76 | 0.0118 |
| GO:0070498~interleukin-1-mediated signaling pathway | 3 | 1.76 | 0.0118 |
| GO:0006959~humoral immune response | 4 | 2.35 | 0.0125 |
| GO:0010629~negative regulation of gene expression | 8 | 4.71 | 0.0130 |
| GO:0060252~positive regulation of glial cell proliferation | 3 | 1.76 | 0.0141 |
| GO:0051726~regulation of cell cycle | 8 | 4.71 | 0.0148 |
| GO:0045672~positive regulation of osteoclast differentiation | 3 | 1.76 | 0.0153 |
| GO:0030097~hemopoiesis | 4 | 2.35 | 0.0155 |
| GO:0009617~response to bacterium | 5 | 2.94 | 0.0157 |
| GO:0051384~response to glucocorticoid | 4 | 2.35 | 0.0161 |
| GO:0009612~response to mechanical stimulus | 4 | 2.35 | 0.0161 |
| GO:0004666~prostaglandin-endoperoxide synthase activity | 2 | 1.18 | 0.0163 |
| GO:1904659~glucose transmembrane transport | 3 | 1.76 | 0.0166 |
| GO:0043154~negative regulation of cysteine-type endopeptidase activity involved in apoptotic process | 4 | 2.35 | 0.0174 |
| GO:0071542~dopaminergic neuron differentiation | 3 | 1.76 | 0.0179 |
| GO:0014070~response to organic cyclic compound | 4 | 2.35 | 0.0181 |
| GO:0030335~positive regulation of cell migration | 7 | 4.12 | 0.0187 |
| GO:0009791~post-embryonic development | 4 | 2.35 | 0.0188 |
| GO:0071385~cellular response to glucocorticoid stimulus | 3 | 1.76 | 0.0192 |
| GO:0036120~cellular response to platelet-derived growth factor stimulus | 3 | 1.76 | 0.0192 |
| GO:0050679~positive regulation of epithelial cell proliferation | 4 | 2.35 | 0.0195 |
| GO:0007568~aging | 6 | 3.53 | 0.0196 |
| GO:0004888~transmembrane signaling receptor activity | 6 | 3.53 | 0.0199 |
| GO:0050728~negative regulation of inflammatory response | 5 | 2.94 | 0.0209 |
| GO:0005887~integral component of plasma membrane | 19 | 11.18 | 0.0216 |
| GO:0007165~signal transduction | 18 | 10.59 | 0.0230 |
| GO:0098869~cellular oxidant detoxification | 4 | 2.35 | 0.0232 |
| GO:0048010~vascular endothelial growth factor receptor signaling pathway | 3 | 1.76 | 0.0234 |
| GO:0001774~microglial cell activation | 3 | 1.76 | 0.0234 |
| GO:0007498~mesoderm development | 3 | 1.76 | 0.0234 |
| GO:0060548~negative regulation of cell death | 4 | 2.35 | 0.0240 |
| GO:0017018~myosin phosphatase activity | 4 | 2.35 | 0.0246 |
| GO:0042327~positive regulation of phosphorylation | 3 | 1.76 | 0.0249 |
| GO:0001934~positive regulation of protein phosphorylation | 6 | 3.53 | 0.0269 |
| GO:0005654~nucleoplasm | 41 | 24.12 | 0.0270 |
| GO:0030593~neutrophil chemotaxis | 4 | 2.35 | 0.0273 |
| GO:0005788~endoplasmic reticulum lumen | 7 | 4.12 | 0.0286 |
| GO:0001503~ossification | 4 | 2.35 | 0.0300 |
| GO:0030522~intracellular receptor signaling pathway | 3 | 1.76 | 0.0312 |
| GO:0090050~positive regulation of cell migration involved in sprouting angiogenesis | 3 | 1.76 | 0.0312 |
| GO:0071409~cellular response to cycloheximide | 2 | 1.18 | 0.0313 |
| GO:0031077~post-embryonic camera-type eye development | 2 | 1.18 | 0.0313 |
| GO:0005737~cytoplasm | 54 | 31.76 | 0.0321 |
| GO:0007155~cell adhesion | 10 | 5.88 | 0.0327 |
| GO:0007626~locomotory behavior | 4 | 2.35 | 0.0327 |
| GO:0007613~memory | 4 | 2.35 | 0.0327 |
| GO:0048662~negative regulation of smooth muscle cell proliferation | 3 | 1.76 | 0.0329 |
| GO:0032570~response to progesterone | 3 | 1.76 | 0.0346 |
| GO:0010467~gene expression | 4 | 2.35 | 0.0346 |
| GO:0071347~cellular response to interleukin-1 | 4 | 2.35 | 0.0346 |
| GO:0070374~positive regulation of ERK1 and ERK2 cascade | 6 | 3.53 | 0.0358 |
| GO:0007267~cell-cell signaling | 6 | 3.53 | 0.0362 |
| GO:0035976~transcription factor AP-1 complex | 2 | 1.18 | 0.0371 |
| GO:0051213~dioxygenase activity | 3 | 1.76 | 0.0387 |
| GO:0043576~regulation of respiratory gaseous exchange | 2 | 1.18 | 0.0390 |
| GO:0002158~osteoclast proliferation | 2 | 1.18 | 0.0390 |
| GO:0002282~microglial cell activation involved in immune response | 2 | 1.18 | 0.0390 |
| GO:0070555~response to interleukin-1 | 3 | 1.76 | 0.0399 |
| GO:0001975~response to amphetamine | 3 | 1.76 | 0.0417 |
| GO:0046627~negative regulation of insulin receptor signaling pathway | 3 | 1.76 | 0.0417 |
| GO:0006955~immune response | 9 | 5.29 | 0.0422 |
| GO:0060070~canonical Wnt signaling pathway | 4 | 2.35 | 0.0429 |
| GO:0097421~liver regeneration | 3 | 1.76 | 0.0436 |
| GO:1901216~positive regulation of neuron death | 3 | 1.76 | 0.0455 |
| GO:0030501~positive regulation of bone mineralization | 3 | 1.76 | 0.0455 |
| GO:0060754~positive regulation of mast cell chemotaxis | 2 | 1.18 | 0.0466 |
| GO:0001541~ovarian follicle development | 3 | 1.76 | 0.0474 |
| GO:0032691~negative regulation of interleukin-1 beta production | 3 | 1.76 | 0.0474 |
| GO:0005172~vascular endothelial growth factor receptor binding | 2 | 1.18 | 0.0482 |
| GO:1990830~cellular response to leukemia inhibitory factor | 4 | 2.35 | 0.0485 |
| GO:0043407~negative regulation of MAP kinase activity | 3 | 1.76 | 0.0494 |
| GO:0032722~positive regulation of chemokine production | 3 | 1.76 | 0.0494 |

**Supplementary Table 2. KEGG pathways of 161 common DEGs with *P* < 0.05**

| **Description** | **ID** | **P-Value** | **Count** |
| --- | --- | --- | --- |
| TNF signaling pathway | hsa04668 | 3.74E-08 | 11 |
| Osteoclast differentiation | hsa04380 | 1.25E-07 | 11 |
| IL-17 signaling pathway | hsa04657 | 7.51E-07 | 9 |
| NF-kappa B signaling pathway | hsa04064 | 1.65E-05 | 8 |
| MAPK signaling pathway | hsa04010 | 1.67E-05 | 13 |
| Rheumatoid arthritis | hsa05323 | 6.59E-05 | 7 |
| AGE-RAGE signaling pathway in diabetic complications | hsa04933 | 0.0001047 | 7 |
| Human T-cell leukemia virus 1 infection | hsa05166 | 0.0001462 | 10 |
| Kaposi sarcoma-associated herpesvirus infection | hsa05167 | 0.0002567 | 9 |
| Cytokine-cytokine receptor interaction | hsa04060 | 0.0003521 | 11 |
| Arachidonic acid metabolism | hsa00590 | 0.0005173 | 5 |
| Viral protein interaction with cytokine and cytokine receptor | hsa04061 | 0.0007627 | 6 |
| Small cell lung cancer | hsa05222 | 0.0032795 | 5 |
| Fluid shear stress and atherosclerosis | hsa05418 | 0.0040910 | 6 |
| Amoebiasis | hsa05146 | 0.0051005 | 5 |
| Breast cancer | hsa05224 | 0.0053717 | 6 |
| Circadian rhythm | hsa04710 | 0.0059494 | 3 |
| Parathyroid hormone synthesis, secretion and action | hsa04928 | 0.0059980 | 5 |
| Cellular senescence | hsa04218 | 0.0071399 | 6 |
| Thyroid cancer | hsa05216 | 0.0075451 | 3 |
| Serotonergic synapse | hsa04726 | 0.0084144 | 5 |
| Bladder cancer | hsa05219 | 0.0100317 | 3 |
| Colorectal cancer | hsa05210 | 0.0144513 | 4 |
| NOD-like receptor signaling pathway | hsa04621 | 0.0160807 | 6 |
| Cocaine addiction | hsa05030 | 0.0162863 | 3 |
| Epstein-Barr virus infection | hsa05169 | 0.0231384 | 6 |
| Legionellosis | hsa05134 | 0.0243021 | 3 |
| Endometrial cancer | hsa05213 | 0.0254293 | 3 |
| C-type lectin receptor signaling pathway | hsa04625 | 0.0270013 | 4 |
| Lipid and atherosclerosis | hsa05417 | 0.0302239 | 6 |
| Th17 cell differentiation | hsa04659 | 0.0304592 | 4 |
| HIF-1 signaling pathway | hsa04066 | 0.0313628 | 4 |
| Basal cell carcinoma | hsa05217 | 0.0314813 | 3 |
| Human cytomegalovirus infection | hsa05163 | 0.0365562 | 6 |
| Wnt signaling pathway | hsa04310 | 0.0384263 | 5 |
| Amphetamine addiction | hsa05031 | 0.0396514 | 3 |
| Renal cell carcinoma | hsa05211 | 0.0396514 | 3 |
| Epithelial cell signaling in Helicobacter pylori infection | hsa05120 | 0.0411081 | 3 |
